# Complete mitochondrial genome of *schistura yingjiangensis* (Zhu 1982) (cypriniformes: nemacheilidae): insights into its features and phylogenetic relationships

**DOI:** 10.1080/23802359.2025.2559714

**Published:** 2025-09-13

**Authors:** Chunxiang Yang, Xiangxing Lu, Guangming Wu, Chunpeng Li, Jianyu Song, Junjie Wu

**Affiliations:** ^a^ Research Center for Aquatic Biotechnology, Yunnan Institute of Fishery Sciences Research, Kunming, China; ^b^ Key Laboratory of Yunnan Characteristic Fish Protection and Germplasm Innovation, Kunming, China; ^c^ Zhaotong City Fisheries Management Station, Zhaotong, China; ^d^ Fish Disease Research Center, Yunnan Institute of Fishery Sciences Research, Kunming, China

**Keywords:** *Schistura yingjiangensis*, mitochondrial genome, phylogenetics, evolutionary biology

## Abstract

This study sequenced and analyzed the complete mitochondrial genome (16,571 bp) of *Schistura yingjiangensis*, a species endemic to Yunnan, China. The genome contains 13 protein-coding genes, 22 tRNAs, and 2 rRNAs. Phylogenetic analysis revealed a close relationship between *S. yingjiangensis, S. polytaenia*, and *S. longa*. This work helps clarify the evolution of the large, paraphyletic genus *Schistura* and contributes to understanding the evolutionary dynamics of Cypriniformes.

## Introduction

Mitochondrial DNA (mtDNA) has been widely used in phylogenetic studies due to its maternal inheritance, high mutation rate, and lack of recombination (Alvarenga et al. 2024). The complete mitochondrial genome provides a comprehensive resource for understanding the evolutionary history and genetic diversity of species (Boore 1999). *Schistura yingjiangensis* (Zhu 1982) is an small endemic freshwater loach distributed in the Mengdian River and Daying River of China, classified to the Cypriniformes, family Nemacheilidae (Zhu 1982; Chen 2013). A decline in the taxonomic diversity of fish assemblages has been observed in the Daying River. In response, the Binlangjiang Aquatic Germplasm Resource Protection Area was established in 2008 to specifically protect fish species in the Daying River, including *S. yingjiangensis* (Cao 2000; Yang et al. 2016). Although the mitochondrial genomes of other *Schistura* have been analyzed in previous studies (Siva et al. 2018), ; Sharma et al. 2020; Peng et al. 2024; the mitochondrial genome of *S. yingjiangensis* has not been previously characterized. Here, we present the complete mitochondrial genome of *S. yingjiangensis* and explore its phylogenomic and evolutionary relationships within the Nemacheilidae.

## Materials and methods

### Sample collection and DNA extraction

**Figure 1.**
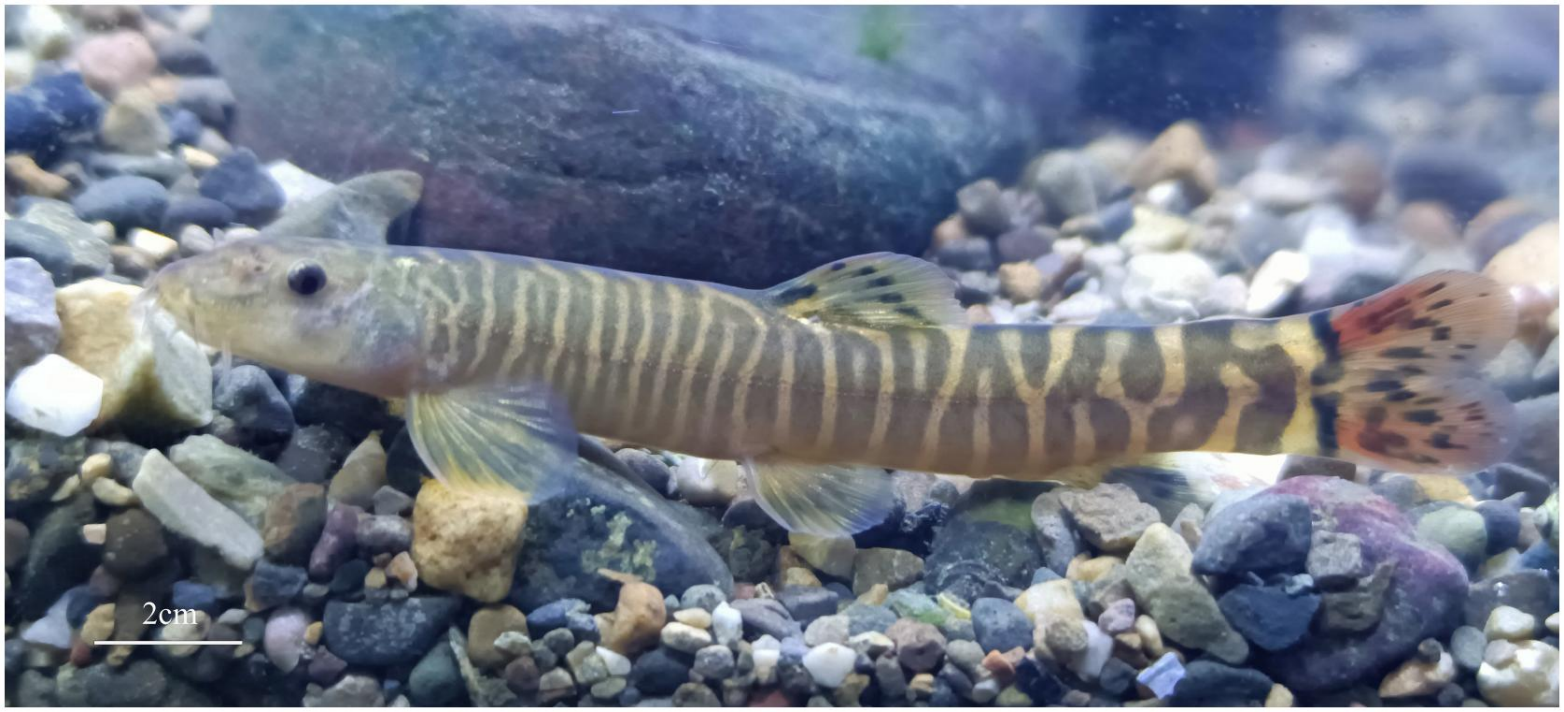
A reference image of *S. yingjiangensis* used in this study was collected by Junjie Wu in the Daying River (N: 24°44′39″, E: 98°3′1″), yingjiang, Yunnan, China. The reference image was photographed by Junjie Wu and Jianyu Song.

The specimens of *S. yingjiangensis* was collected in Yingjiang River (N: 24°44′39′′, E: 98°3′1′′), Yingjiang, Yunnan, China. The status of the collected specimens was recorded, comprising 3 live individuals. The three live specimens ranged from 20.6 to 22.7 cm in total length (TL) and 17.9 to 19.5 cm in standard length (SL). Total genomic DNA was extracted from muscle tissue using TIANamp Genomic DNA Kit (TIANGEN Co., Ltd, Beijing, China) following the manual’s protocol. The quality and quantity of the DNA were assessed using agarose gel electrophoresis and spectrophotometry. Three samples were deposited in the Yunnan Institute of Fishery Sciences Research (contact person: Chunxiang Yang, miehuoqi1995@outlook.com) under voucher number YJNQ-01-03. The descriptive image of a sample was captured by Junjie Wu and Jianyu Song (corresponding authors) (Figure 1).

### Mitogenome sequencing and assembly

Genomic DNA was extracted from muscle tissue using the TIANamp Genomic DNA Kit (TIANGEN Co., Ltd, Beijing, China) following the manufacturer’s protocol. The abundance and quality of DNA were assessed using the NanoPhotometer^®^ N60 spectrophotometer. The Illumina library was constructed following the manufacturer’s protocols with the TruSeq^™^ Nano DNA Sample Preparation Kit (Illumina, U.S.) (Meyer and Kircher 2010). Subsequently, the NovaSeq platform was employed to generate raw sequencing reads (Modi et al. 2021), with the sequencing services provided by Tsingke Co., Ltd. (Beijing, China). The raw reads were assembled using MitoZ 3.6 (Meng et al. 2019), and the mitogenome was annotated using MITOS with the default parameters (Bernt et al. 2013).

### Phylogenetic analysis

The phylogenetic relationship of *S. yingjiangensis* was inferred by using the 13 protein-coding gene (PCGs) sequences from 63 related species. Multiple sequence alignment was performed using MAFFT with default parameters (Katoh et al. 2019), and phylogenetic trees were constructed using the maximum likelihood (ML) methods as implemented in IQ-TREE with the an effective stochastic algorithm and 5000 bootstrap duplications (Nguyen et al. 2015; Kalyaanamoorthy et al. 2017).

## Results

### Mitogenome organization and composition

**Figure 2.**
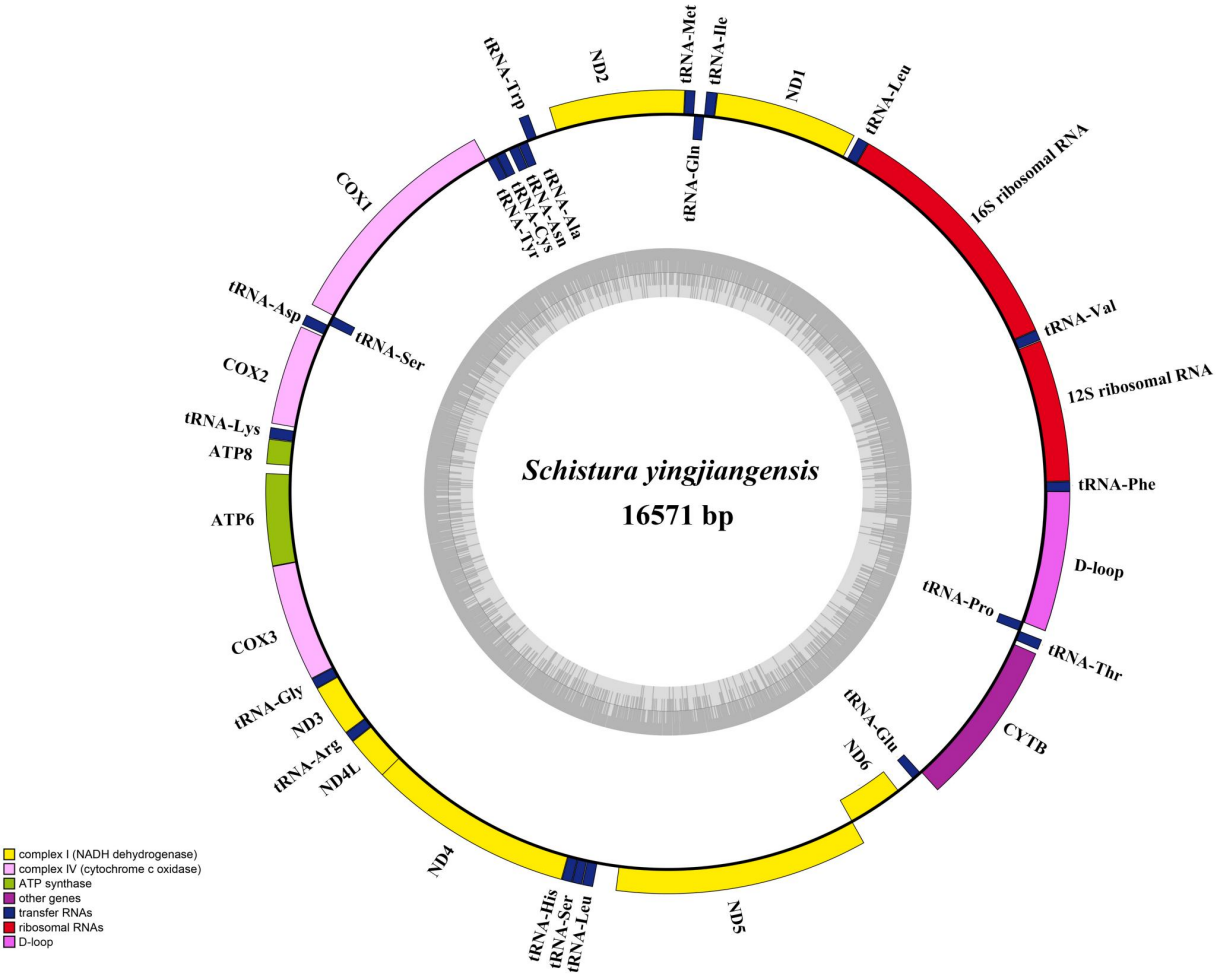
The complete mitochondrial genome map of *S. yingjiangensis* (GenBank: PP114298) is presented. Encoded genes and RNAs are depicted in different colors. The light and heavy strands are shown on the inner and outer sides of the circle, respectively.

The complete mitochondrial genome of *S. yingjiangensis* is 16571 bp in length with a depth of 678.9x (Supplementary Figure 1) and exhibits the typical circular structure found in vertebrates. It contains 13 PCGs, 22 tRNA genes, 2 rRNA genes, and a control region (D-loop). The overall base composition is 30.82%A, 24.92%T, 16.51%G, 27.75%C, with an AT skew. Among the 13 protein-coding genes (PCGs), 12 initiated with the start codon ATG, while *COI* is transcribed with GTG. Six PCGs (*ND1*, *ND4L*, *ND5*, *ND6*, *COI*, and *ATP8*) had complete stop codons (TAA). Incomplete stop codons were identified in *ND2*, *ND3*, *ND4*, *ATP6*, *COII*, *COIII*, and *CYTB*. Additionally, 12 PCGs (excluding *ND6*) and 14 tRNA genes were encoded on the heavy strand (Figure 2). The gene order and orientation are consistent with other fish species in the genus *Schistura*.

### Phylogenetic analysis

**Figure 3.**
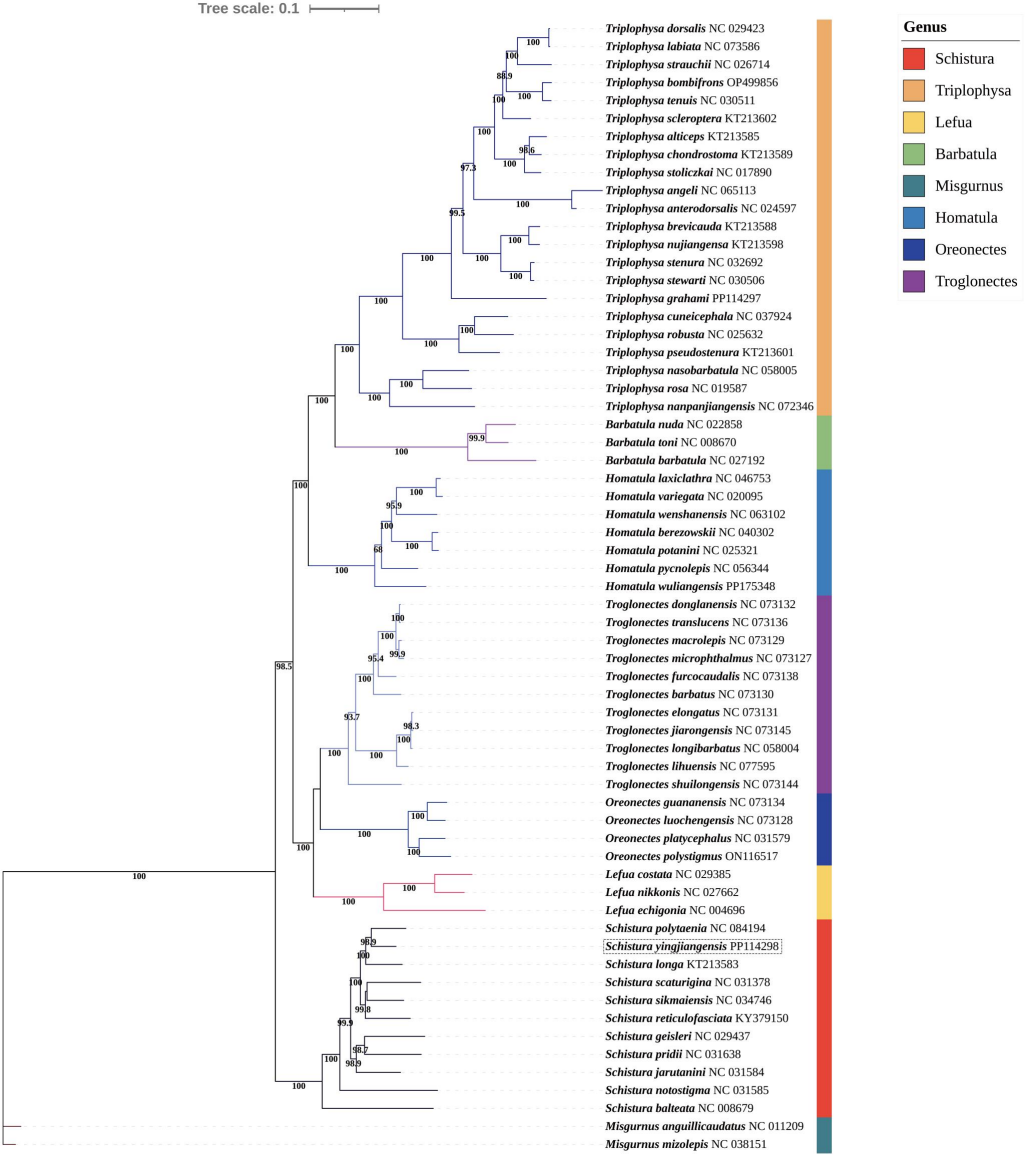
Phylogenetic tree of *S. yingjiangensis* and other 62 species based on the complete mitogenomes from NCBI. *Misgurnus anguillicaudatus* and *misgurnus bipartitus* were treated as outgroups. The bootstrap values were marked near the nodes. Accession numbers for each species were listed following the name of the species. The following sequences were used: *B.barbatula* (NC 027192, (Murienne et al. 2016)), *B. nuda* (NC 022858, (Zhao et al. 2015)), *B. toni* (NC 008670, (Saitoh et al. 2006)), *T. alticeps* (KT213585, (Wang et al. 2016)), *T. chondrostoma* (KT213589, (Wang et al. 2016)), *T. stoliczkai* (NC 017890, (Li et al. 2013)), *T. bombifrons* (OP499856), *T. tenuis* (NC 030511), *T. dorsalis* (NC 029423, (Lei et al. 2016)), *T. labiata* (NC 073586), *T. strauchii* (NC 026714, (Kanu et al. 2016)), *T. scleroptera* (KT213588, (Wang et al. 2016)), *T. nujiangensa* (KT213598, (Wang et al. 2016)), *T. stenura* (NC 032692), *T. stewarti* (NC 030506), *T. grahami* (PP114297), *T. cuneicephala* (NC 037924), *T. robusta* (NC 025632, (Yan et al. 2016)), *T. pseudostenura* (KT213601, (Wang et al. 2016)), *T. nanpanjiangensis* (NC 072346), *T. nasobarbatula* (NC 058005), *T. rosa* (NC 019587, (Wang et al. 2012)), *H. berezowskii* (NC 040302), *H. potanini* (NC 025321, (Que et al. 2016)), *H. laxiclathra* (NC 046753), *H. vatiegata* (NC 020095), *H. wenshanensis* (NC 063102), *H. pycnolepis* (NC 056344), *H. wuliangensis* (PP175348), *L. costata* (NC 029385), *L. nikkonis* (NC 027662, (Miya et al. 2015)), *L.echigonia* (NC 004696, (Saitoh et al. 2003)), *O.guananensis* (NC 073134, (Luo et al. 2023)), *O. luochengensis* (NC 073128, (Luo et al. 2023)), *O. platycephalus* (NC 031579), *O. polystigmmus* (ON116517, (Luo et al. 2023)), *T. barbatus* (NC 073130, (Luo et al. 2023)), *T. donglanensis* (NC 073132, (Luo et al. 2023)), *T. translucens* (NC 073136, (Luo et al. 2023)), *T. macrolepis* (NC 073129, (Luo et al. 2023)), *T. microphthalmus* (NC 073127, (Luo et al. 2023)), *T. furcocaudalis* (NC 073138, (Luo et al. 2023)), *T. elongatus* (NC 073131, (Luo et al. 2023)), *T. jiarongensis* (NC 073145, (Luo et al. 2023)), *T. longibarbatus* (NC 058004), *T. lihuensis* (NC 077595), *T. shuilongensis* (NC 073144, (Luo et al. 2023)), *S. balteata* (NC 008679, (Saitoh et al. 2006)), *S.geisleri* (NC 029437), *S. pridii* (NC 031638), *S. jarutanini* (NC 031584), *S. longa* (KT213583, (Wang et al. 2016)), *S. polytarnia* (NC 084194), *S. reticulofasciata* (KY379150), *S. scaturigina* (NC 031378), *S. sikmaiensis* (NC 034746 (Sharma et al. 2020)), *S. notostigma* (NC 031585), *M. anguillicaudatus* (NC 011209 (He et al. 2008)), *M. mizolepis* (NC 038151 (Lee 2016)).

The maximum likelihood (ML) tree revealed that all members of the genus *Schistura* formed a monophyletic group, such as *S. balteata*, *S. geisleri*, *S. pridii*, *S. jarutanini*, *S. longa*, *S. polytaenia*, *S. reticulofasciata*, *S. scaturigina*, *S. sikmaiensis*, and *S. notostigma*, were clustered within the *Schistura* clade, with strong bootstrap support. Phylogenetic analysis indicated that *S. yingjiangensis* is closely associated with the *Schistura* clade that includes *S. polytaenia* and *S. longa* (Figure 3). These findings offer novel perspectives on the evolutionary relationships within the Nemacheilidae family.

## Discussion

In this study, The mitogenome of *S. yingjiangensis* exhibits high conservation in gene order, base composition, and AT bias (55.74% AT content), consistent with the characteristics of most freshwater fish mitogenomes (Wang et al. 2016; Siva et al. 2018;). Notably, the *COI* gene in *S. yingjiangensis* starts with GTG, while other PCGs use ATG as the initiation codon, a feature shared with other *Schistura* species such as *S. fasciolata*, *S. sikmaiensis* and *S. reticulofasciata* (Siva et al. 2018; Sharma et al. 2020; Peng et al. 2024; Additionally, the incomplete stop codons ‘T’ in *ND2*, *ND3*, *ND4*, *ATP6*, *COII*, *COIII*, and *CYTB* genes are likely completed to TAA through post-transcriptional polyadenylation, a common phenomenon in fish mitogenomes (Broughton et al. 2001).

Phylogenetic analysis reveals that the evolutionary relationship of *S. yingjiangensis* is close to *S. polytaenia* and *S. longa*, which corroborates the results of previous study (Peng et al. 2024). These findings contribute vital genetic data crucial for elucidating the evolution and taxonomy within the genus *Schistura*.(Wang et al. 2016)

## Conclusion

This study presents the first complete mitogenome of *S. yingjiangensis*, enriching the *Schistura* genetic database and providing valuable insights into the species’ phylogenetic relationships, evolutionary biology, and conservation, thereby contributing to the broader understanding of fish mitochondrial genome evolution and laying a foundation for future research.

## Supplementary Material

Supplemental Material

## Data Availability

The complete mitochondrial genome assembly data of *S. yingjiangensis* was available in GenBank database under the accession number PP114298, the associated BioProject, BioSample and SRA numbers are PRJNA1123854, SAMN41829188 and SRR29409023, respectively.
