# Supplementary figures and images for: Complete mitochondrial genome of schistura yingjiangensis (Zhu 1982) (cypriniformes: nemacheilidae): insights into its features and phylogenetic relationships
Source: Mitochondrial DNA B Resour. 2025 Sep 13;10(10):937–41. doi: 10.1080/23802359.2025.2559714 (PMC12434845; doi:10.1080/23802359.2025.2559714)

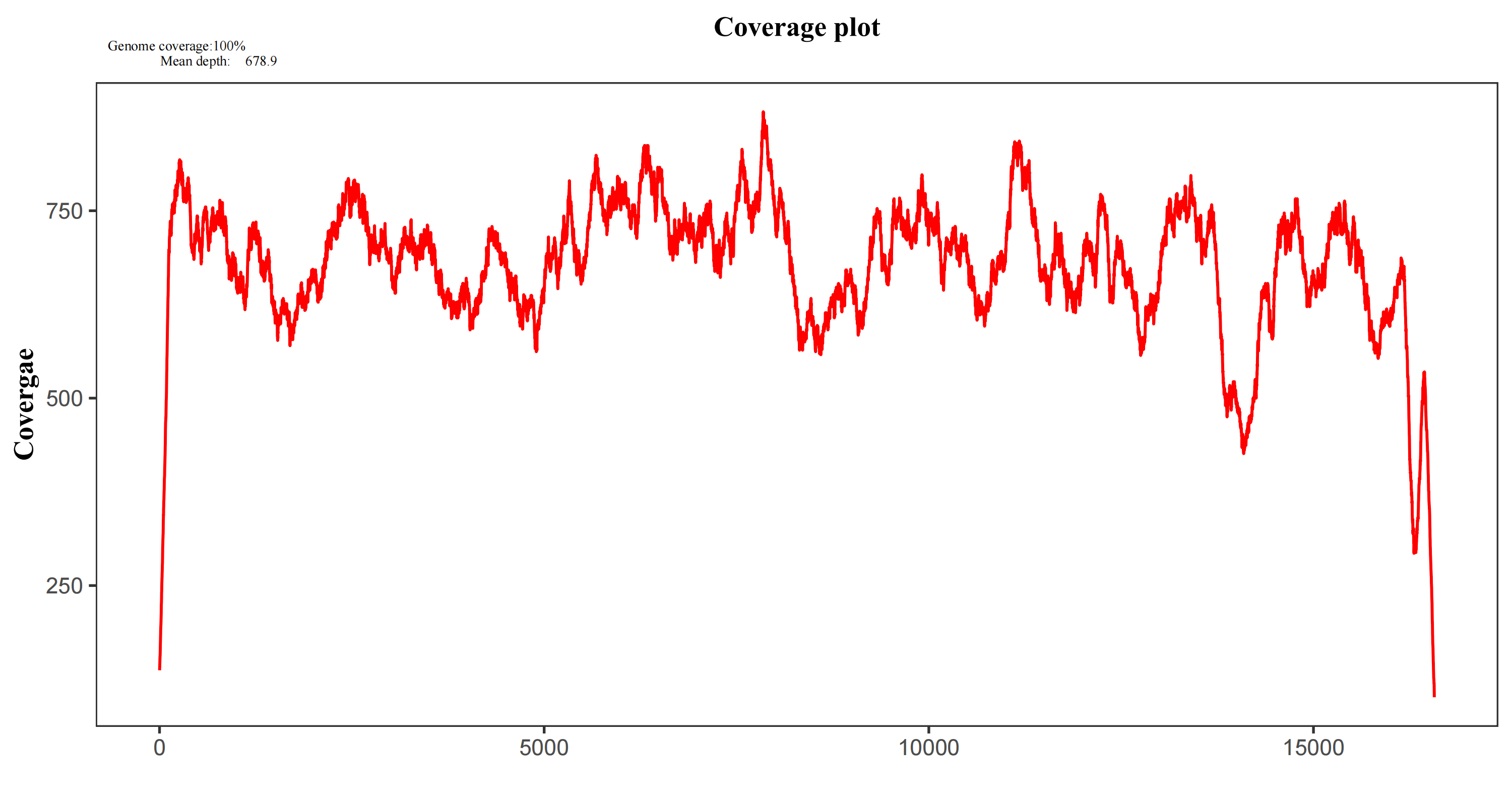

Supplement: Supplemental Material [file TMDN_A_2559714_SM2245.tif]
